# Influence of Ternary Emulsifier Mixtures on Oxidative Stability of Nanoemulsions Based on Avocado Oil

**DOI:** 10.3390/foods9010042

**Published:** 2020-01-03

**Authors:** Natalia Riquelme, Camila Sepúlveda, Carla Arancibia

**Affiliations:** 1 Food Science and Technology Department, Technological Faculty, Universidad de Santiago de Chile, Obispo Umaña 050, Estación Central 9170201, Chile; natalia.riquelme@ug.uchile.cl (N.R.); camila.sepulveda@usach.cl (C.S.); 2 Food Science and Chemical Technology Department, Universidad de Chile, Santos Dumont 964, Independencia 8380494, Chile

**Keywords:** emulsifier mixtures, nanoemulsions, oxidative stability, avocado oil

## Abstract

The aim of this work was to study the effect of two emulsifiers (M_1_: SL-soy lecithin, Tw80-Tween 80 and CasCa-calcium caseinate and M_2_: SL-soy lecithin, Tw80-Tween 80 and SE-sucrose esters) on the oxidative stability of avocado oil-based nanoemulsions. Oil-in-water nanoemulsions were prepared using 3.6% *w*/*w* of two emulsifier mixtures, which were optimized by mixture experimental design in order to minimize particle size (PS) and polydispersity index (PdI). Then, the oxidative stability of nanoemulsions was evaluated through both an induction period and a quantification of hydroperoxides and thiobarbituric acid reactive species (TBARs) under accelerated storage conditions. The simplex-centroid mixture design showed that PS and PdI varied when proportions of different emulsifiers were modified, obtaining an optimized concentration for each mixture of: 85% SL, 10% Tw80 and 5%CasCa (M_1_) and 85% SL, 7.4% Tw80 and 7.6% SE (M_2_) that produced nanoemulsions with PS ~116 nm and PdI < 0.2. Nanoemulsions elaborated with M1 and M2 presented similar particle characteristics and physical stability to the control sample with Tw80. However, M1 nanoemulsions were more stable against lipid oxidation, since they showed the highest induction period and lower formation of hydroperoxides and TBARs during storage.

## 1. Introduction

In the last decade the interest in the development of food products based on avocado oil has increased markedly, since this oil is a good source of bioactive compounds such as antioxidant vitamins, phystosterols, α-tocopherol, β-carotene, lutein and oleic acid (~70%) corresponding to the ω-9 family [1,2]. In addition, several studies have reported that avocado oil could reduce the risk of cardiovascular diseases and improve the lipid profile of patients with moderate hypercholesterolemia [1]. However, the most edible oils are chemically unstable and susceptible to lipid oxidation, especially when are exposed to oxygen, light, moisture and temperature [3].

Lipid oxidation is one of the main factors that affect the quality and shelf life of food products because of the formation of primary oxidation products (hydroperoxides), which are then decomposed to carbonyls and other compounds, in particular aldehydes during storage. Lipid oxidation negatively influences sensory quality, leading to changes in flavor, texture and color, and nutrition of food products [4]. Oil-in-water (O/W) food emulsions are also susceptible to oxidative degradation, since the lipid phase can interacts with the pro-oxidants present in the aqueous phase, such as transition metals, enzymes, and photosensitizers [5]. Hence, lipid oxidation is highly dependent on interfacial characteristics and composition [6]. In addition, the greater surface area of oil droplets in nanoemulsions (particle sizes- PS < 100 nm) increases the rate of lipid oxidation, because of greater contact between oil droplets and the aqueous phase [7]. However, this is not so clear since other studies have observed an opposite behavior [8,9], where emulsions with the largest particle size (3–6 μm) have showed a greater formation of hydroperoxides and thiobarbituric acid reactive substances (TBARs).

Emulsifying agents are used to facilitate the formation of nanoemulsions and ensure their physical long-term stability [10], because they are molecules with surface activity that are quickly absorbed onto the oil-water interface. These reduce the interfacial tension and generate repulsive interactions between the oil droplets, which allows an improved stability during storage [11]. Emulsifiers may also positively impact chemical stability of nanoemulsions, because of the molecular interactions among them or with other emulsifiers at the aqueous phase or at the lipid droplet surfaces, which may causing a change in composition and structure of oil-water interface leading to changes in the physical and chemical stability of nanoemulsions [4]. Therefore, the type of emulsifiers used for stabilizing nanoemulsions can be fundamental in the maintenance of oxidative stability since they can vary the size and conformation of oil-water interface, specially their thickness, permeability, charge, structure and composition [12,13].

In the same way, the emulsifier can also be key to improve the oxidative stability of nanoemulsions due to its antioxidant effect, since they can controlling the ability of prooxidants, free radicals and oxygen to interact with oil droplets, delaying lipid oxidation [14]. Accordingly, it is possible to design an effective interfacial layer to inhibit lipid oxidation of O/W nanoemulsions by selecting a suitable emulsifier. Several studies have evaluated the effect of the molecular weight of synthetic emulsifiers on the oxidative stability of o/w nanoemulsions [15,16], and the influence of emulsifier nature -synthetic or natural- comparing the effectiveness of different surfactants (quillaja saponins, Tween 80 and soy lecithin) in the maintenance of nanoemulsions oxidative stability [17,18]. In addition, a few studies [19,20,21] provides the advantage of the oxidative stability of nanoemulsions being improved in comparison with using a single emulsifier. For example, they improve the oxidative stability of nanoemulsions in comparison with a single emulsifier. In particular, the study conducted by Yi et al. [6] demonstrated that the use of binary emulsifiers mixture (sodium caseinate-Tween 20) decreases lipid oxidation in emulsions (monitored by hydroperoxides and TBARs formation), in contrast to those produced with a single emulsifier (Tween 20). Similarly, [22] reported that the mixture of sodium caseinate and phosphatidylcholine improved oxidative stability of high fat fish oil emulsions. They observed significantly lower 1-penten-3-ol production because of the enhancement of interfacial properties at the oil-water emulsion interface. However, the use of ternary mixtures of emulsifiers to improve chemical stability of O/W nanoemulsions has not been relatively little studied. In a previous study, we studied the effect of ternary emulsifiers mixture (soy lecithin, Tween 80 and quillaja saponins) on physical stability of O/W nanoemulsions under different processing conditions (ionic strength and temperature) [23]. Our results showed that ternary emulsifier mixtures were more efficient than binary ones at maintaining physical characteristics and thermal stability; however, its stability against lipid oxidation was not evaluated. In this context, the purpose of this work was to study the effect of two ternary emulsifier mixture on oxidative stability of O/W nanoemulsions.

## 2. Materials and Methods

### 2.1. Materials

Nanoemulsions were prepared with purified water from an inverse osmosis system (Vigaflow, Santiago, Chile), avocado oil (Casta de Peteroa, Terramater, Santiago, Chile), and emulsifier mixtures composed of soy lecithin (Metarin P, Cargill, Blumos S.A., Santiago, Chile), polysorbate 80 (Tween 80, Sigma-Aldrich, Saint Louis, MO, USA), calcium caseinate (ProtLight IP4, Armor Proteines, Blumos S.A., Santiago, Chile) and sucrose esters (SP70, Sisterna, Roosendaal, The Netherlands). For oxidative stability tests, several analytical grade reagents were used: butanol, isooctane, isopropanol, methanol, cumene hydroperoxide, ammonium thiocyanate, barium chloride, iron sulfate, trichloroacetic acid, thiobarbituric acid and malonaldehyde. These were purchased from Merck (Santiago, Chile).

### 2.2. Experimental Design and Emulsion Preparation

Oil-in-water nanoemulsions were prepared using 3% *w*/*w* of avocado oil and 3.6% *w*/*w* of a mixture of emulsifiers (1:1, oil:emulsifier ratio), which was optimized by Surface Response Methodology using an experimental mixture design. Mixture 1 (M_1_) was composed of soy lecithin-SL, Tween 80-Tw80 and calcium caseinate-CasCa, and Mixture 2 (M_2_) by soy lecithin-SL, Tween 80-Tw80 and sucrose ester-SE. The optimization of emulsifier mixture proportions (experimental variables) was realized using a simplex-centroid mixture experimental design with 10 runs, as shown in Table 1. This type of design allows an optimization according to the relative proportion of the different components, where the different proportions are denoted as $X_{1},X_{2},\ldots ,X_{q}$ following the restrictions: $0\leq X_{i}\leq 1$ for each component i, so that $\sum_{i=1}^{q}X_{i}=1$ [24,25]. Particle size and polydispersity index were evaluated as response variables.

Nanoemulsions were produced in three stages: (1) The continuous phase was prepared dissolving emulsifier mixtures in purified water using magnetic stirring (Arex, Velp Scientifica, Usmate Velate, Italy) at 650 rpm for 40–100 min, depending on the type of emulsifier. (2) A pre-emulsion was formed by adding lipid phase (avocado oil) to the continuous phase using a high-speed homogenizer (T25 Ultraturrax, IKA, Staufen, Germany) at 10,000 rpm for 15 min, and (3) In order to obtain particles sizes at nanometric scale, the pre-emulsion was homogenized through ultrasound (VCX500, Sonics, Newtown, CT, USA) for 30 min, at 20 kHz, 80% of amplitude, and in a pulse mode of 15s on and 10s off. The homogenization process was carried out under an ice bath to prevent over processing phenomenon by overheating. Once the nanoemulsions were obtained, they were stored in glass containers at 5 °C for 24 h before being analyzed.

### 2.3. Nanoemulsion Characterization

#### 2.3.1. Particle Size and Polydispersity Index

Particle size (PS) and polydispersity index (PdI) of nanoemulsions were determined using dynamic light scattering (DLS) instrument (NanoS90, Malvern Instruments, Malvern, UK). Before the measurement, nanoemulsions were diluted with miliQ water until a clear solution was obtained (concentration of 8% *v*/*v* approx.) and then deposited in a standard measuring cell. Refraction indices of 1.47 for lipid phase (avocado oil) and 1.33 for continuous phase (purified water) were used, and the refractive index of the protein was assumed to be 0 [26]. The reported values of PS and PdI corresponded to an average of three measurements.

#### 2.3.2. Zeta Potential

Zeta potential (ZPot) of nanoemulsions was determined by Electrophoretic Light Scattering (ELS) using a Zetasizer (Nano-ZS, Malvern Instruments, Malvern, UK). For this purpose, nanoemulsions were diluted at 8% *v*/*v* in milli-Q water and deposited in capillary cells equipped with two electrodes (Disposable folded capillary cell, Malvern Instruments, Malvern, UK). ZPot values were collected over 30 continuous readings and all samples were measured in triplicate.

#### 2.3.3. Physical Stability

The physical stability of nanoemulsions was evaluated by creaming index (CI). Aliquots of 15 mL of each nanoemulsion were placed in conical centrifuge tubes to carry out a centrifugation process at 2400× *g* for 15 min (Universal 32R, Hettich, Tuttlingen, Germany) in order to accelerate destabilization of nanoemulsion [27]. The creaming index was calculated by Equation (1) and after the centrifugation process:

(1) $$CI\left(\%\right)=\frac{H_{S}}{H_{E}}\times 100$$

where, $H_{E}$ is the total height of the nanoemulsion and $H_{S}$ is the height of the cream layer formed.

### 2.4. Oxidative Stability

Oxidative stability was monitored by the determination of: induction time, and both primary (hydroperoxide) and secondary (TBARs) compound formation. To evaluate oxidative stability during storage time, nanoemulsions were stored in sealed plastic tubes under accelerated conditions at 50 °C for 20 days. Measurements were realized for 0, 5, 10, 15 and 20 days.

#### 2.4.1. Induction Period Time

A rapid oxidative stability tester (RapidOxy, Anton Paar, Graz, Austria) was used to determine differences in the oxidative-induction period of nanoemulsions. This equipment accelerates the oxidation process by increasing the temperature and oxygen pressure of an equipment chamber, where the sample is introduced and into which oxygen is pumped at a fixed pressure. Oxygen consumption is marked as the pressure drops, where the induction period corresponds to the time taken to cause a defined pressure drop equals 10% [28]. This measuring principle is based on ASTM- D8206 (Standard Test Method for Oxidation Stability of Lubricating Greases—Rapid Small-Scale Oxidation Test), which measures the net change in pressure resulting from consumption of oxygen by oxidation and gain in pressure due to formation of volatile oxidation by-products. To perform measurements, 5 mL of each nanoemulsions were placed in a plastic plate (40 mm diameter) and incubated at 120 °C and 700 kPa in the chamber of the equipment. Each measurement was performed in triplicate and the results were reported as mean and standard deviation.

#### 2.4.2. Quantification of Hydroperoxides

Lipid hydroperoxide formation during storage was measured using an adaptation of the methodology proposed by [29]. Lipid extraction from the samples was carried out by adding 0.3 mL of each nanoemulsion to 1.5 mL of a mixture of isooctane:isopropanol (3:1% *v*/*v*), followed by vortexing at 24,000 rpm for 10 s (3 times) and centrifugation (MiniSpin Plus, Eppendorf, Hamburg, Germany) at 3000 rpm for 2 min. Then, 0.1 mL supernatant was mixed with 2.8 mL methanol: butanol (2:1% *v*/*v*), 15 μL 3.94 M ammonium thiocyanate, and 15 μL ferrous iron solution, which was prepared by mixing equal amounts of 0.13 M barium chloride and 0.14 M iron sulfate. The absorbance at 510 nm was measured in a spectrophotometer (UV Mini 1240, Shimadzu, Kyoto, Japan) after 20 min of storage in the dark at room temperature. Hydroperoxide concentrations were determined using a cumene hydroperoxide standard curve at concentrations from 0 to 5.5 mM. All nanoemulsions were measured in triplicate.

#### 2.4.3. Thiobarbituric Acid-Reactive Substances (TBARS)

Secondary oxidation products were determined by TBARs method [30]. 50 μL of each nanoemulsion was mixed with 2 mL of the TBA (thiobarbituric acid) solution (15% *w*/*v* trichloroacetic acid, 0.375% *w*/*v* TBA in 0.25 M HCl) and 1 mL of distilled water. After that, the mixtures were placed in a thermoregulated bath at 90 °C (B-100, Buchi, Flawil, Switzerland) for 15 min, cooled at room temperature and then centrifuged at 3400 rpm for 15 min. The absorbance of the supernatant was measured in a microplate reader (Multiscan Go, Thermo Scientific, Vantaa, Finland) at 580 and 532 nm. The final absorbance was calculated as the difference between the absorbance measured at 532 and 580 nm (A532-A580), where A580 represents the scattering of light produced by non-TBARs species, eliminating interference from other compounds [31]. Concentrations of TBARs were determined from a standard curve prepared from a solution of 1,1,3,3-tetraethoxypropane with an increasing concentration from 0 to 1 mM.

#### 2.4.4. Statistical Analysis

Surface Response Methodology (RSM) and an experimental mixture design were used to optimize response variables (particle size and polydispersity index). The experimental design data were adjusted to a special cubic model as is described in Equation (2):

(2) $$Y=\sum_{i=1}^{q}\beta_{i}X_{i}+\sum_{i<j}\sum_{j=2}^{q}\beta_{ij}X_{i}X_{j}+\sum_{i<j}\sum_{j<k}\sum_{k=3}^{q}\beta_{ijk}X_{i}X_{j}X_{k}$$

where, Y is the response variable; i,j and k are the number of emulsifiers in the mixture; $\beta_{i}$ is the first-order coefficient, $\beta_{ij}$ is the second-order coefficient, $\beta_{ijk}$ is the third-order coefficient; $X_{i}$, $X_{j}$ and $X_{k}$ are the emulsifiers concentrations in the mixture.

Experimental design data were analyzed using a level of significance of α = 0.05, and the optimum proportions of each mixture emulsifier were determined using STATGRAPHICS software (Centurion XVI, Warrenton, VA, USA).

An analysis of variance (ANOVA) was carried out on experimental data from physical characterization and oxidative test using XLSTAT software (version 2015 17.1, Addinsoft, Paris, France).

## 3. Results & Discussion

### 3.1. Optimization of Emulsifiers Proportions

Two mixtures of emulsifiers were studied according to a simplex-centroid mixtures experimental design. The purpose was to determine the optimal proportions of ternary emulsifier mixture that allow the formation of nanoemulsions with a smaller particle size (PS < 150 nm) and polydispersity index (PdI < 0.2).

#### 3.1.1. Ternary Mixture 1 (M_1_): Soy Lecithin, Tween 80 and Sodium Caseinate

**Figure 1:**
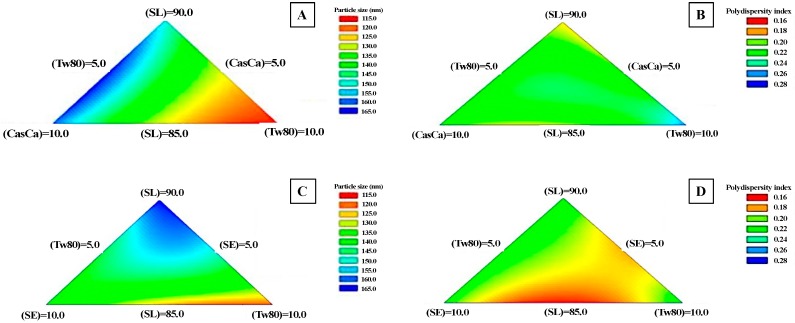
Mixture contour plots for particle size and polydispersity index of nanoemulsions elaborated with different emulsifier mixtures, (**A**,**B**): Mixture 1 (soy lecithin—SL, Tween 80—Tw80 and sodium caseinate—CasCa) and (**C**,**D**): Mixture 2 (soy lecithin—SL, Tween 80—Tw80 and sucrose ester—SE).

Table 2 shows results (particle size and polydispersity index) for nanoemulsions produced with the M1 mixture. In the case of particle size (PS), the values decreased significantly (*p* < 0.05) at the highest concentration of Tween 80 (10%) (118.6 and 114.4 nm to run 3 and 10, respectively), whilst polydispersity index (PdI) values varied slightly from 0.17 to 0.21, where a high concentration of soy lecithin (90%) produced nanoemulsions with the highest PdI values (~0.2 for run 1 and 6). To establish binary or ternary interactions of the experimental factors (proportions of emulsifiers), the particle size and polydispersity index data were adjusted to a special cubic model, which was significant (*p* < 0.05) and explained 95% and 89% of the variability contained in the respective response variables. In addition, the fit of the model did not show a significant effect (*p* > 0.05) of the binary and ternary interactions between experimental factors to PS variable, whilst only the binary interaction between CasCa and Tw80 had a significant effect (*p* < 0.05) on the PdI values. The contour plots of the PS and PdI are shown in Figure 1A,B, respectively. To PS, at low concentrations of SL and CasCa and at high levels of Tw80, PS values < 120 nm were obtained (warm tone zone of Figure 1A). In the case of PdI response, most of the combinations of emulsifiers formed nanoemulsions with a PdI < 0.2. At the lowest concentrations of SL (85%) and CasCa (5%) and at high concentration of Tw80 (10%), PdI values ~0.17 were obtained (yellow-orange zone of Figure 1B), which could indicate a good physical stability of nanoemulsions during storage [32]. 

Finally, a multiple optimization of the experimental factors was carried out to obtain the optimum emulsifier concentrations in order to minimize both response variables, particle size and polydispersity index. The optimal combination of the different experimental factors (emulsifiers proportions) corresponded to 85% SL, 5% CasCa and 10% Tw80, predicting optimal PS and PdI values equal to 116.5 nm and 0.172, respectively.

#### 3.1.2. Ternary Mixture 2 (M_2_): Soy Lecithin, Tween 80 and Sucrose Ester

For the emulsifiers mixture M_2_, the experimental results of the response variables (PS and PdI) are shown in Table 2. As in the Mixture 1, the lowest PS values were obtained at the highest concentrations of Tw80 (10%) (111.8 and 116.5 nm for run 3 and 10, respectively); however, at intermediate levels of SL (7.5%) and Tw80 (7.5%) (run 6), PS values close to 116 nm were also observed, which suggests a synergism between both emulsifiers in the formation of nanoemulsions that produced small PS of oil droplets. Regarding PdI, slightly larger PdI values were observed with respect to Mixture 1 (among 0.18–0.22 nm), although at the medium levels of SE (7.5%) and Tw80 (7.5%), the lowest PdI values (PdI = 0.167) were obtained (run 6). The experimental results of PS and PdI were adjusted to a special cubic model that established a significant (*p* < 0.05) relationship between the experimental factors and the response variables, which explained 96% and 99% of the variability contained in the PS and PdI, respectively. No significant (*p* > 0.05) interactions (binary and ternary) among the experimental factors on PS variable were found, although a significant effect (*p* < 0.05) of binary interactions (SL-Tw80 and SE-Tw80) and ternary interaction (SL-Tw80-SE) on PdI variable was observed. Figure 1C,D show the contour plots for PS and PdI response variables, respectively. In general, when the concentration of Tw80 increased, PS values decreased until ~112 nm, which is observed in Figure 1C (yellow-orange zone). Conversely, at high concentrations of SL (90%) and lower concentrations of SE (5%) and Tw80 (5%), PS values close to 144 nm were obtained (green-blue zone in Figure 1C). In the case of the PdI response, it was observed that at the lowest levels of SL the smallest values of PdI (PdI = 0.167) were obtained (orange-red zone in Figure 1D). In order to obtain optimum surfactant concentrations of M2 that minimize PS and PdI, a multiple optimization of experimental variables was carried out, where the optimal combination of the different experimental factors (emulsifier concentrations) corresponded to 85% SL, 7.4% SE and 7.6% Tw80, predicting values of 120.8 nm and 0.209 to PS and PdI, respectively.

### 3.2. Nanoemulsion Characterization

#### Particle Characteristics and Physical Stability

Two control samples were prepared to compare ternary emulsifiers mixtures (M1 and M2) efficiency in contrast to a single emulsifier: C_Tw80_ of Tween 80 and C_SL_ of soy lecithin. Table 3 shows the average values of particle size, polydispersity index, and zeta potential of different nanoemulsions. Controls C_Tw80_ and C_SL_ showed the lowest and highest PS values (72.4 and 214.6 nm) respectively. The ternary emulsifiers mixtures (M1 and M2) produced significantly lower PS values (*p* < 0.05) than the control of natural emulsifier (CSL), but they were less efficient than the synthetic emulsifier control (C_Tw80_) in decreasing PS (Table 3). The good emulsifying capacity of Tw80 is already known, because these molecules can migrate quickly to the oil-water interface, decreasing the interfacial tension [18,33]. This favors the generation of smaller oil droplets during the homogenization process [34]. When comparing ternary mixtures, no significant differences (*p* > 0.05) were found in the PS, where values of 115.0 and 119.5 nm for M_1_ and M_2_ were obtained, respectively (Table 3). In the case of PdI, control samples showed an opposite effect, since C_SL_ presented the lowest PdI value (0.17), while C_Tw80_ the highest value (0.28), and emulsifiers mixtures (M_1_ and M_2_) intermediate values (0.19 and 0.21, respectively). Notably, PdI < 0.2 is an indicator of homogeneous distribution of particle size and good physical stability of nanoemulsions [35], so that nanoemulsions produced with the ternary emulsifier mixture can remain stable during storage.

Regarding zeta potential, all nanoemulsions presented a negative electric charge (Table 3). The control nanoemulsion with soy lecithin was the most electronegative one (−52.8 ± 0.9 mV), because this emulsifier has a negative charge which is attributable to the presence of anionic phospholipids [35]. Conversely, the control sample with Tween 80 was the least electronegative one (−14.3 ± 1.3 mV). Tw80 is a non-ionic surfactant, but a considerable negative charge was observed in the control sample with Tween 80 (Table 3), which may be because of the oil or surfactant contained anionic impurities (such as free fatty acids) or because of preferential adsorption of anions (such as hydroxyl ions) from the water phase [6]. No significant differences (*p* > 0.05) were found between nanoemulsions prepared with emulsifier mixtures, where ZPot values were observed to be close to the C_SL_ sample because of the high soy lecithin concentration in the emulsifier mixture.

**Figure 2:**
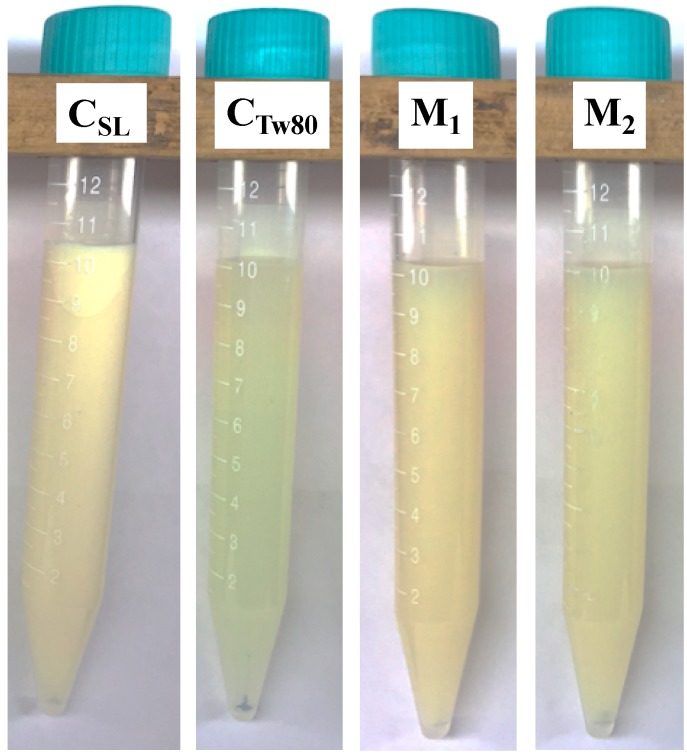
Physical stability of nanoemulsions elaborated with different emulsifier mixtures and control samples after the centrifugation process at 2400× *g* for 15 min. M_1_: mixture 1 (soy lecithin—SL, Tween 80—Tw80 and sodium caseinate—CasCa), M_2_: mixture 2 (soy lecithin—SL, Tween 80—Tw80 and sucrose ester—SE), C_SL_: control soy lecithin, and C_Tw80_: control Tween 80.

Physical stability of nanoemulsions was studied by creaming index, which was determined after a centrifugation process at 2400× *g* for 15 min. Figure 2 shows nanoemulsion photographs after the centrifugation process, where the formation of cream layer was only observed in the control sample prepared with soy lecithin (C_SL_), a creaming index percentage (%CI) of 10% (Table 3). 

In the case of the other nanoemulsions (M_1_, M_2_ and C_Tw80_), no creaming formation was observed. These results can be related with the particle characteristics of these nanoemulsions, since at PS < 200 nm there is an increase in Brownian force. This force favors the homogeneous distribution of the droplets in the nanoemulsions, reducing the influence of gravitational forces, which can improve the physical stability of the nanoemulsions [36]. In addition, the zeta potential (ZPot) of M_1_ and M_2_ samples was >30 (absolute value), possibly indicating good physical stability during storage [10]. However, C_SL_ also presented ZPot values >|30|, but was destabilized by creaming after centrifugation process (Figure 2, Table 3), indicating that PS is the most influential characteristic on physical stability of these type of nanoemulsions.

### 3.3. Oxidative Stability

**Figure 3:**
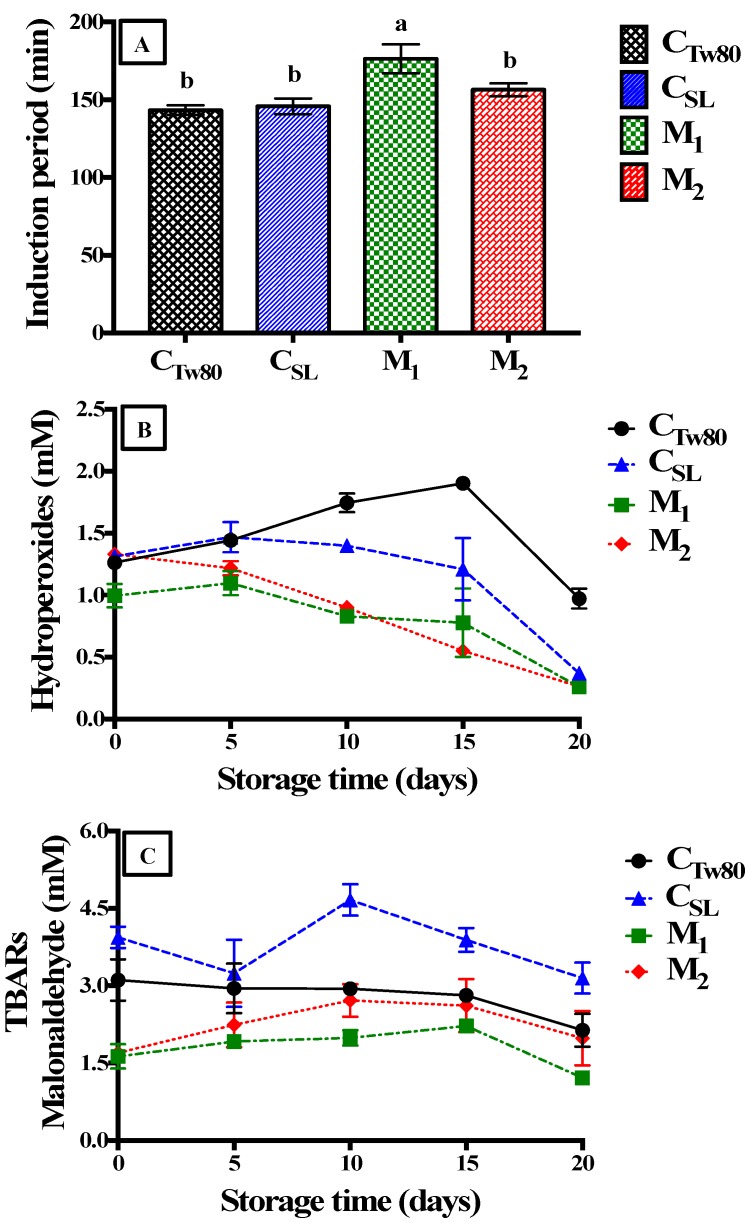
Oxidative stability of nanoemulsions elaborated with different emulsifiers mixtures and control samples: induction period (**A**), primary oxidation (**B**) and secondary oxidation (**C**). M_1_: mixture 1 (soy lecithin—SL, Tween 80—Tw80 and sodium caseinate—CasCa), M_2_: mixture 2 (soy lecithin—SL, Tween 80—Tw80 and sucrose ester—SE), C_SL_: control soy lecithin, and C_Tw80_: control Tween 80. Different letters indicate significant differences (*p* < 0.05) in the induction period for different samples.

Oxidative stability of nanoemulsions was evaluated in three stages: induction period, primary oxidation and secondary oxidation. Figure 3A shows the induction period for all nanoemulsions studied, where it is observed that the nanoemulsion produced with M_1_ presented a significantly higher induction time (176.4 min, *p* < 0.05) than the other samples, which indicated a higher stability against lipid oxidation. 

This difference can be explained as a consequence of the composition of the interface of this system (M_1_). Here, proteins (such as CasCa)—molecules of larger size than other surfactants (SL, Tw80 or SE)—can generate a dense protective interface layer, promoting a steric interference that prevents the contact of prooxidant substances present in the aqueous phase with the lipid phase [5]. On the other hand, control samples (C_Tw80_ and C_SL_) showed a lower induction time (143.3 and 145.8 min, respectively) than nanoemulsions with ternary emulsifier mixtures (Figure 3A). In the case of Tw80, the lower induction time can be caused by the formation of a thin layer of surfactant molecules that promote the diffusion of prooxidants from the aqueous phase to the oil droplets [36]. The control sample with soy lecithin, in contrast, was less stable, probably because of its highly negative charge that attracts positively charged transition metals to their oil droplet surfaces, increasing the oxidation rate [37].

Regarding hydroperoxides, in the first days of storage an increase of hydroperoxides concentration was observed in most of the nanoemulsions studied (M_1_, C_SL_ and C_Tw80_) (Figure 3B). Later, a few of them also increased at the final storage time, while the nanoemulsion with the emulsifier mixture M_2_ showed a rapid decrease of hydroperoxides (from day 5) probably because of the rapid formation of secondary products of lipid oxidation. On the other hand, control samples (C_SL_ and C_Tw80_) showed a higher formation of hydroperoxides during storage time in relation to M_1_ and M_2,_ because both surfactants did not create dense interfaces or a steric interference that could avoid the contact between oil droplets and prooxidants present in the aqueous phase [38]. The nanoemulsion with emulsifier mixture M_1_ presented the lowest hydroperoxides formation during storage time probably because of the chelating capacity of CasCa, or the steric interference of proteins at the interface [5] that protected the oil droplets from oxidation during storage. In the case of M_2_, nanoemulsions was less efficient

In the case of secondary oxidation, the nanoemulsions produced with the emulsifiers mixtures (M_1_ and M_2_) showed the lowest formation of thiobarbituric reactive substances (TBARs) after 20 days of storage at 50 °C (Figure 3C). This confirms the fact that emulsifier mixtures can delay lipid oxidation because of the interactions between emulsifiers at the interface or the formation of emulsifier multilayers that protect oil droplets [12]. These results are in agreement with other studies, where it was observed that binary emulsifier mixtures (protein and small surfactant molecules) can improve lipid oxidation. García-Moreno et al. [38] reported that the use of sodium caseinate and soybean lecithin mixture improves the oxidative stability of fish oil emulsions, and they suggested that the combination of these emulsifiers forms a favorable structure and thickness in the interfacial layer that prevent its lipid oxidation. Yi et al. [6] studied the effect of different proportions of sodium caseinate and Tween 20 on oxidative stability of emulsions based on walnut oil, and they found that the highest proportions of sodium caseinate retard lipid oxidation since Tween 20 cannot displace sodium caseinate from the droplet surfaces, improving its chelating properties. In the case of M_2_, it was observed that mixture with sucroester (SE) was less efficient than one with CasCa to prevent the formation of TBARs, but this mixture was better than a single emulsifier, which it can be due to the formation of mixed micelles between Tween 80 and SE that are adsorbed at the oil-water interfase protecting oil droplets against to lipid oxidation [15]. Regarding control samples, C_SL_ presented the highest formation of TBARs (Table 3), probably because of the emulsifier composition, since soy lecithin is composed of mixtures of phospholipids that can oxidize during storage. Conversely, C_Tw80_ remained stable during storage, but showed a higher TBARs concentration in comparison with M_1_ y M_2_. Thus, the use of ternary emulsifier mixtures reduces lipid oxidation of nanoemulsions, which is useful for the manufacturing of food products based on emulsions that encapsulate lipid bioactive compounds.

## 4. Conclusions

The use of emulsifier mixtures allows obtaining nanoemulsions with particle size near 100 nm and to improve their physical stability, in comparison with nanoemulsions produced only with soy lecithin; however, its effectiveness on reducing particle size and maintaining physical stability is similar to the control with Tween 80. Emulsifier mixtures are more efficient on maintaining the oxidative stability of nanoemulsions than single emulsifiers. Here in, the mixture composed by soy lecithin, Tween 80 and calcium caseinate, presented the lowest formation of primary and secondary oxidation compounds. Thus, it can be concluded that the use of emulsifier mixtures in the production of nanoemulsions can help to maintain their oxidative and physical stability during the storage, which can be useful for the development of emulsions-based delivery systems enriched in omega fatty acids.

## Figures and Tables

**Table 1:** Experimental factors, levels and response variables for mixture experimental design.

Experimental Factors (Emulsifier Mixture)	Levels (%)		Response Variables
	Low	High	
**Mixture 1 (M_1_)**			Particle size (nm) Polydispersity index
Soy lecithin (SL)	85	90	
Tween 80 (Tw80)	5	10	
Sodium caseinate (CasCa)	5	10	
**Mixture 2 (M_2_)**			
Soy lecithin (SL)	85	90	
Tween 80 (Tw80)	5	10	
Sucrose esters (SE)	5	10	

The total concentration of emulsifiers mixture was fixed at 3.6% *w*/*w* for both emulsifier mixtures.

**Table 2:** Particle size and polydispersity index of nanoemulsions correspondent to each run of experimental design for two emulsifier mixtures studied.

Run Number	Emulsifier Concentration (%)			Particle Size (nm)	Polydispersity Index
M_1_	SL	Tw80	CasCa		
1	90	5	5	154.4 ± 6.0 ^bc^	0.209 ± 0.006 ^a^
2	85	5	10	161.9 ± 6.7 ^ab^	0.198 ± 0.005 ^b^
3	85	10	5	118.6 ± 7.1 ^fg^	0.166 ± 0.005 ^e^
4	87.5	5	7.5	163.4 ± 3.4 ^a^	0.182 ± 0.003 ^d^
5	87.5	7.5	5	128.7 ± 7.3 ^e^	0.193 ± 0.005 ^bc^
6	85	7.5	7.5	127.6 ± 6.7 ^ef^	0.200 ± 0.005 ^ab^
7	86.6	6.7	6.7	137.8 ± 8.2 ^de^	0.174 ± 0.004 ^de^
8	90	5	5	147.2 ± 4.4 ^cd^	0.186 ± 0.004 ^cd^
9	85	5	10	156.6 ± 2.9 ^ab^	0.179 ± 0.009 ^d^
10	85	10	5	114.4 ± 1.6 ^g^	0.177 ± 0.002 ^de^
**M_2_**	**SL**	**Tw80**	**SE**		
1	90	5	5	142.2 ± 3.2 ^A^	0.221 ± 0.005 ^A^
2	85	5	10	131.4 ± 4.2 ^B^	0.211 ± 0.002 ^AB^
3	85	10	5	111.8 ± 3.8 ^C^	0.188 ± 0.011 ^CD^
4	87.5	5	7.5	130.9 ± 4.3 ^B^	0.199 ± 0.006 ^BC^
5	87.5	7.5	5	134.5 ± 3.7 ^B^	0.188 ± 0.008 ^CD^
6	85	7.5	7.5	116.8 ± 3.8 ^C^	0.167 ± 0.004 ^E^
7	86.7	6.7	6.7	133.6 ± 3.6 ^B^	0.188 ± 0.007 ^CD^
8	90	5	5	144.4 ± 3.8 ^A^	0.213 ± 0.007 ^AB^
9	85	5	10	129.9 ± 6.3 ^B^	0.197 ± 0.010 ^BC^
10	85	10	5	116.5 ± 3.3 ^C^	0.176 ± 0.005 ^DE^

M_1_: mixture 1 (soy lecithin-SL, Tween 80-Tw80 and sodium caseinate-CasCa), and M_2_: mixture 2 (soy lecithin—SL, Tween 80—Tw80 and sucrose ester—SE). Different letters indicate significant differences (*p* < 0.05) in the same parameter (column) in each emulsifier mixture.

**Table 3:** Physical properties of nanoemulsions elaborated with different emulsifier mixtures and control samples elaborated with a single emulsifier.

Sample	Particle Size (nm)	Polydispersity Index	Zeta Potential (mV)	Creaming Index (%)
**C_SL_**	214.6 ± 1.3 ^c^	0.173 ± 0.008 ^a^	−52.8 ± 0.9 ^c^	10 ± 1
**C_Tw80_**	72.4 ± 3.1 ^a^	0.284 ± 0.009 ^c^	−14.3 ± 1.3 ^a^	n.o.
**M_1_**	115.0 ± 2.1 ^b^	0.199 ± 0.009 ^b^	−43.1 ± 1.8 ^b^	n.o.
**M_2_**	119.5 ± 3.8 ^b^	0.205 ± 0.011 ^b^	−45.6 ± 0.4 ^b^	n.o.

M_1_: mixture 1 (soy lecithin-SL, Tween 80-Tw80 and sodium caseinate-CasCa), M_2_: mixture 2 (soy lecithin-SL, Tween 80-Tw80 and sucrose ester-SE), C_SL_: control with soy lecithin, and C_Tw80_: control with Tween 80. n.o.: no creaming formation observed. Different letters indicate significant differences (*p* < 0.05) in the parameters (same column) for different samples.
